# Construction and Manipulation of Serial Gradient Dilution Array on a Microfluidic Slipchip for Screening and Characterizing Inhibitors against Human Pancreatic Lipase

**DOI:** 10.3390/bios13020274

**Published:** 2023-02-15

**Authors:** Junqiang Yang, Yanyan Deng, Min Zhang, Shilun Feng, Sheng Peng, Shijia Yang, Peirong Liu, Gaozhe Cai, Guangbo Ge

**Affiliations:** 1 Department of Anesthesiology, Seventh People’s Hospital of Shanghai University of TCM, Shanghai 200137, China; 2 Shanghai Frontiers Science Center of TCM Chemical Biology, Institute of Interdisciplinary Integrative Medicine Research, Shanghai University of Traditional Chinese Medicine, Shanghai 201203, China; 3 Key Laboratory of Xinjiang Phytomedicine Resource and Utilization, Ministry of Education, Pharmacy School of Shihezi University, Shihezi 832099, China; 4 State Key Laboratory of Transducer Technology, Shanghai Institute of Microsystem and Information Technology, Chinese Academy of Sciences, Shanghai 200050, China; 5 Department of Anesthesiology, Longhua Hospital Shanghai University of TCM, Shanghai 200032, China

**Keywords:** microfluidic, SlipChip, serial gradient dilution, obesity, human pancreatic lipase, inhibitors

## Abstract

Obesity is one of the foremost public health concerns. Human pancreatic lipase (hPL), a crucial digestive enzyme responsible for the digestion of dietary lipids in humans, has been validated as an important therapeutic target for preventing and treating obesity. The serial dilution technique is commonly used to generate solutions with different concentrations and can be easily modified for drug screening. Conventional serial gradient dilution is often performed with tedious multiple manual pipetting steps, where it is difficult to precisely control fluidic volumes at low microliter levels. Herein, we presented a microfluidic SlipChip that enabled formation and manipulation of serial dilution array in an instrument-free manner. With simple slipping steps, the compound solution could be diluted to seven gradients with the dilution ratio of 1:1 and co-incubated with the enzyme (hPL)-substrate system for screening the anti-hPL potentials. To ensure complete mixing of solution and diluent during continuous dilution, we established a numerical simulation model and conducted an ink mixing experiment to determine the mixing time. Furthermore, we also demonstrated the serial dilution ability of the proposed SlipChip using standard fluorescent dye. As a proof of concept, we tested this microfluidic SlipChip using one marketed anti-obesity drug (Orlistat) and two natural products (1,2,3,4,6-penta-O-galloyl-β-D-glucopyranose (PGG) and sciadopitysin) with anti-hPL potentials. The IC_50_ values of these agents were calculated as 11.69 nM, 8.22 nM and 0.80 μM, for Orlistat, PGG and sciadopitysin, respectively, which were consistent with the results obtained by conventional biochemical assay.

## 1. Introduction

Obesity continues to be among the foremost public health concerns of the 21st century, sweeping invariably through both developed and developing countries, with the number of overweight and obese adults reaching 1.9 billion and 650 million, respectively [1,2,3,4]. Obesity is widely recognized as a disorder of lipid metabolism, and targeting the enzymes involved in this process can lead to the development of highly effective anti-obesity drugs [5,6,7]. As the crucial lipolytic enzyme synthesized and secreted by the pancreas, human pancreatic lipase (hPL) is a predominant anti-obesity target, responsible for the regulation of lipid digestion and absorption in vivo [8,9,10]. Thus, hPL inhibitors can reduce the hydrolysis of dietary lipids in the gastrointestinal tract by decreasing the hydrolytic activity of hPL, thereby alleviating the symptoms of obesity and overweight [11,12,13]. Orlistat (tetrahydrolipstatin, THL), a product of Streptomyces toxitrycini, which is endorsed by food and drug administrations, has been demonstrated to be a highly specific inhibitor of human pancreatic lipase through covalent binding to serine (the active site of the enzyme) [11]. In addition, countless therapeutic strategies have been developed over the years, especially in the realm of natural products [14,15,16,17]. In our past studies, we identified that 1,2,3,4,6-penta-*O*-galloyl-β-D-glucopyranose (PGG), a bioactive constituent of the root-extract of *Rhodiola crenulata* (ERC), displayed very potent anti-hPL activity, with IC_50_ value of 7.59 nM [18]. Meanwhile, we found that a biflavone component isolated from *Ginkgo biloba* leaves, sciadopitysin, also showed an efficacious inhibitory effect on hPL, with an IC_50_ value of 0.75 μM [15,19].

Serial gradient dilution is a common technique used to generate solutions with gradient concentrations from relatively high-concentration solutions and can be used to set up conditions for drug screening or characterizing IC_50_ values of drugs. However, conventional serial gradient dilution is mostly performed in tubes by tedious multiple manual pipetting, where it is difficult to precisely control fluidic volumes at low microliter volumes [20,21]. Furthermore, the fluorescence-based assay for detecting the IC_50_ of inhibitor on hPL in microplate readers often requires a preparation of incubation systems (total volume: 100 μL) and mixing in tubes before transferring to a 96-well plate [16,18], which leads to a large consumption of reagents and unstable dilution results due to manual operation difference. Therefore, there is a need to develop a lower-cost and easy to operate serial gradient dilution method for drug screening.

Microfluidic devices have been demonstrated to be an excellent tool to control and manipulate small volumes and are widely used in screening tests, with the advantages of high throughput and less sample consumption [22,23,24,25]. Therefore, by utilizing the merits of microfluidic devices, quantitative and reproducible serial dilution can be easily achieved. The typical microfluidic serial dilution method uses a gradient-making network, which was first proposed by Whitesides et al. [26,27] and has been improved and extended for many applications [28,29]. Supplied with two source solutions, the gradient-making network can deliver two solutions with a linear sequence of concentrations. Approaches based on magnetic or electronic liquid manipulation have shown to enable serial dilution with highly flexible dilution ratios [30,31]. Furthermore, centrifugal microfluidic platforms have also been shown to achieve fully automated serial dilution with large dilution ratios [32]. However, those microfluidic devices usually involve complicated equipment manufacturing and operation processes, and require bulky and expensive peripheral accessories, such as various pressure pumps, valves, and precise fluid control systems. The microfluidic SlipChip device [33] is an ideal choice to meet the requirements of on-chip serial dilution in a simple and convenient way. The SlipChip device can switch the required path for fluid operation through the sliding operation of two microfluidic plates with micro holes and channels printed in close contact, without pumps, valves and precise fluid control instruments [34]. Serial dilution has been successfully demonstrated using a self-partitioning SlipChip device for phenotypic antimicrobial susceptibility [35]. In this work, gradient droplets were generated by a surface tension-driven self-partitioning process with a simple slipping step. Although it produced a serial dilution over a large dynamic range, a long diluting time (3 h) was required as the serial dilution entirely depended on the diffusion of the source solution. Additionally, the complex process for the glass treatment to fabricating the glass-based SlipChip may also limit its application. 

**Figure 1:**
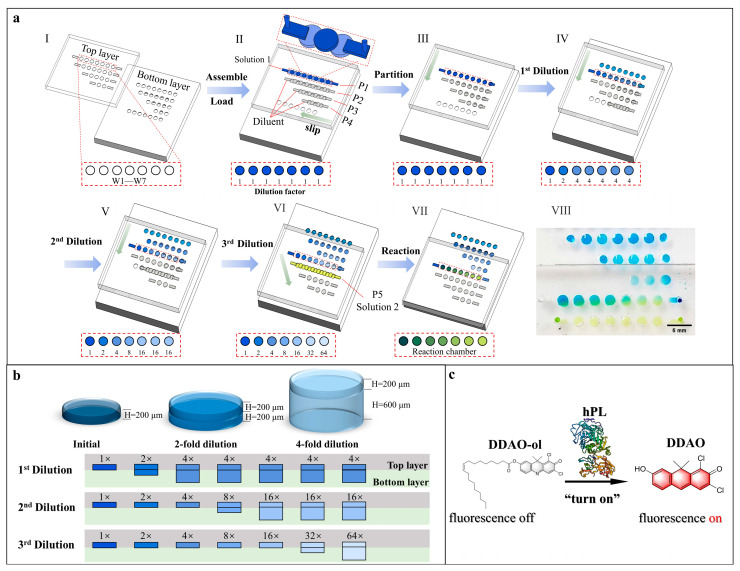
(**a**) Schematic drawings for microfluidic SlipChip operations: (I) top and bottom layers of the SlipChip device; (II) assembly of the SlipChip by partially overlapping the microchambers and ducts on the contacting surface of two layers, original solution (solution 1) and dilution buffer introduced into (P1, P2, P3, P4) channels, respectively; (III) top layer moved left to form isolated reaction partitions; (IV) top layer moved down to perform the 1st dilution step; (V) top layer moved down to perform the 2nd dilution step; (VI) top layer moved down to perform the 3rd dilution step, solution 2 was introduced into (P5) channel; (VII) top layer moved right down to make concentration gradient solution 1 and solution 2 react in chambers; (VIII) photo of the reaction of serial diluted solution 1 (blue ink) and solution 2 (yellow ink) in the chamber. (**b**) Zoom-in view of the assembly of the top and the bottom layer microchambers, and schematic diagram of initial concentration, 2-fold dilution and 4-fold dilution. (**c**) Principle of the enzymatic reaction between hPL and its substrate.

In this paper, we proposed a portable, low-cost, and easy to operate microfluidic polydimethylsiloxane (PDMS)-based SlipChip. As shown in Figure 1, the proposed SlipChip was composed of two PDMS layers with microchambers and fluidic ducts on the contact surface. The top PDMS layer consisted of four rows of microchambers for solution injection and the bottom PDMS layer consisted of five rows of microchambers with different heights (200 μm and 600 μm) and the same diameter (2 mm) for multiple dilution. By simply sliding the chip in sequence, a serial gradient dilution array of the regent (solution 1) with seven concentrations at a dilution ratio of 1:1 was formed. The formed serial gradient dilution array on the top layer can be reacted with another regent (solution 2) on the bottom layer by sliding again to align two layers. As a proof of concept, the inhibitory effect of Orlistat, PGG, and sciadopitysin on the hPL enzyme-catalyzed reaction was tested using this proposed microfluidic SlipChip. The results fully complied with the results of previous experiments using 96-well plates and commercial microplate readers. This microfluidic SlipChip device can provide a simple and promising research platform for drug concentration characterization in vitro and can also be used for research in the field of Chinese herbal medicine. Moreover, it has a broad application prospect in other biochemical detection fields.

## 2. Materials and Methods

### 2.1. Materials and Reagents

Silicone oil with a viscosity of 10,000 cSt, rhodamine fluorescent dye (Rhodamine 6G) and phosphate-buffered saline (PBS) were all purchased from Sigma-Aldrich (St. Louis, MO, USA). The silicone elastomer kit for fabricating the polydimethylsiloxane (PDMS) chip was purchased from Dow Corning (Sylgard 184, Auburn, MI, USA). The specific substrate (DDAO-ol) was synthesized in our laboratory. Bile salt was obtained from the Dalian Meilun Biotech Co., Ltd. (Dalian, China). Tris was purchased from Roche Diagnostics GmbH (Mannheim, Germany). Tris-HCl buffer (pH 7.4, 25 mM, 150 mM NaCl, 1 mM CaCl_2_) was prepared by using Milli-Q Water (Millipore, Danvers, MA, USA). Orlistat was purchased from the Dalian Meilun Biotech Co., Ltd. (Dalian China). 1,2,3,4,6-penta-*O*-galloyl-β-D-glucopyranose (PGG) was provided from Jiangyin Tianjiang Pharmaceutical Co., Ltd. (Jiangyin, China). Sciadopitysin was ordered from Shanghai Standard Biotech Co., Ltd. (Shanghai, China). Dimethyl sulfoxide (DMSO) was purchased from Thermo Fisher Scientific (Fair Lawn, NJ, USA).

### 2.2. Fabrication of the Microfluidic SlipChip

The proposed SlipChip was composed of two PDMS layers with microchambers and fluidic ducts on the contact surface and a silicone oil middle layer. The top PDMS layer consists of four rows of microchambers (diameter: 2 mm, height: 200 μm) and ducts (3 mm × 1 mm × 0.2 mm) for solution injection. The bottom PDMS layer consists of five rows of microchambers with different heights (200 μm and 600 μm) and the same diameter (2 mm). Detailed design parameters can be found in Supplementary Materials Figure S2 and Table S1. The mold of this microfluidic chip was designed using Solidworks software (Concord, MA, USA) and fabricated by the Formlabs Form3 3D printer (Somerville, MA, USA). Next, the PDMS prepolymer and the curing agent were mixed at a mass ratio of 10:1 for 10 min and cast in the mold after degassing in vacuum for 30 min, followed by curing at 60 °C overnight. After that, the PDMS layer was peeled from the mold and punched at the inlet and outlet of the top layer (hole diameter: 1.5 mm). Due to good biocompatibility, silicone oil (with a viscosity of 10,000 cst) was used as a lubricant between two PDMS layers to promote chip sliding and prevent liquid leakage. Detail information of the chip fabrication can be found in the Supplementary Material (Figure S1). Briefly, the development of the silicone oil layer was formed by stamping the bottom PDMS layer on a silicone oil film spun on a silicon wafer at 3000 rpm for 50 s. After assembling with top PDMS layer, the SlipChip was then placed horizontally for 10 min to make the silicone oil layer uniform. Finally, the microfluidic chip was slid to connect the microchambers of the two PDMS layers with the fluid pipeline to form four continuous fluid ducts for solution injecting. 

### 2.3. Gradient Dilution Principle and Practical Operation Process of SlipChip

The main principle of the serial gradient dilution in this proposed SlipChip was based on solutions mixing between two chambers of different heights. As shown in Figure 1b, the dilution ratio was 1:1 when a chamber with height of 200 μm in the top layer (chamber_200_top_) was aligned with a chamber with a height of 200 μm in the bottom layer (chamber_200_bottom_). Besides, the dilution ratio was 1:3 when chamber_200_top_ was overlapped with the chamber with a height of 600 μm in the bottom layer (chamber_600_bottom_).

As shown in Figure 1a, after assembling the SlipChip with silicone oil, the top and bottom PDMS layer were at an initial phase (phase II): the four rows of microchambers and fluidic ducts on the top layer and the four rows of microchambers on the bottom layer partially overlapped to establish four continuous fluidic paths (P1, P2, P3, P4)**,** except for the last row of microchambers in the bottom layer. Solution 1 was then introduced into the P1 fluidic path by pipetting, and a solution containing dilution buffer was loaded into the (P2, P3, P4) fluidic paths. Then, the top layer was first slid to the left relative to the bottom layer to disconnect all the fluidic paths and partition reagents (phase III). To perform the 1st dilution, the top layer was moved downward relative to the bottom layer to allow the first row of microchambers on the top plate to overlap with the second row of microchambers on the bottom layer (phase IV). According to the serial dilution principle mentioned above, the solution in the first chamber on the left in top layer remained at the original concentration as there was no chamber overlapped with it in the bottom layer. The solution in the second chamber of the top layer was diluted at the ratio of 1:1 as the chamber_200_top_ and chamber_200_bottom_ overlapped, while the solution in all remaining chambers in the top layer was diluted four times as the chamber_200_top_ and chamber_600_bottom_ were overlapped. The 1st serial dilution factors of the solution in top layer chambers (from left to right) were 1, 2, 4, 4, 4, 4, and 4, respectively. Following the 1st dilution, the 2nd dilution (phase V) and 3rd dilution (phase IV) were performed with the same sliding direction to finally achieve the serial gradient dilution of the solution at a ratio of 1:1 in seven chambers. 

Meanwhile, at phase VI, the second row of microchambers and fluidic paths on the top layer and the fifth row of microchambers on the bottom layer partially overlapped to establish a new continuous fluid path (P5). After pipetting out the residual diluent in P5, solution 2 was then loaded into P5. Finally, the top layer was slipped down and to the right to allow the serial gradient dilution array to mix with the isolated solution 2 in the chambers (phase VII). To demonstrate the serial gradient dilution effect of this proposed microfluidic SlipChip, blue dye, pure water, and yellow dye were loaded into the SlipChip and diluted according to the operation method mentioned above. The collated photo is shown in Figure 1a (VIII).

### 2.4. Optimize the Mixing Time of the Solution

The key factor in the serial gradient dilution is the mixing between solution and diluent. To ensure uniform mixing of the solutions when the chambers on the PDMS layers were overlapped during sliding and serial gradient dilution, the mixing time was first characterized. The prepared blue dye (as solution 1) and the PBS (0.01 M, as diluent) were injected into the SlipChip according to the operation described in Section 2.3. After simple sliding steps, a serial blue dye array with gradient concentrations were established in the microchambers. To optimize the mixing time of high-concentration solution and diluent in the microchambers of the microfluidic SlipChip, we selected four mixing time points: 30 s, 1 min, 3 min, and 5 min. Additionally, we prepared a standard gradient concentration of blue ink solution with a dilution ratio of 1:2 in the centrifuge tube as the control group. The control group was also injected into the SlipChip without dilution for comparison. All photos of the serial blue dye array with gradient concentrations in microchambers were taken with camera under the same conditions. The color information of these pictures and the saturation of the entire area of each well were analyzed and calculated by ImageJ software. Finally, the mixing time was optimized by comparing with the control group.

### 2.5. Fluorescent Dilution Verification Test

To verify the serial dilution ability of this SlipChip for fluorescence detection, a standard fluorescent solution was utilized for evaluating its ability for fluorescent dilution. According to the operation mentioned in Section 2.3, a solution containing rhodamine fluorescent dye solution at a concentration of 400 mg/L was introduced into the P1 fluidic path by pipetting, and DI water was loaded into the (P2, P3, P4) fluidic paths. With sliding steps, a series of microchambers containing fluorescent dye solution with gradient concentrations was established. At the final step, the top layer chambers were slid and overlapped with the bottom layer chambers and continued to slide down to separate from the bottom layer chambers for better photos, which meant that each solution in the top layer chamber at the phase VII; would be 2-fold diluted for imaging. Then the SlipChip device was placed under the fluorescence microscope (Olympus Corporation, Tokyo 163-0914, Japan) with the same fluorescent condition to obtain the pictures of each chamber. The gray value of the fluorescent pictures was analyzed by ImageJ software.

### 2.6. The Screening Process of the Inhibitors Using SlipChip 

Fluorescence-based assays were used to determine the residual activities of hPL in the presence of different concentrations of inhibitor, and to calculate the IC_50_ of the inhibitors. First, Orlistat, PGG, and sciadopitysin were diluted with DMSO to 250 nM, 300 nM, and 20 μM as the initial concentrations, respectively. Then, the inhibitor at the initial concentration was injected into the P1 fluidic path, followed by injecting DMSO into the P2, P3 and P4 fluidic paths. After sliding the SlipChip as described in Section 2.3 with optimized conditions, seven microchambers containing inhibitors with gradient concentrations were formed. At the same time, the enzyme-substrate system was prepared by mixing 111 μL Tris-HCl (pH 7.4, 25 mM), 80 μL bile salts (0.1 mg/mL, final concentration), 1 μL hPL (dissolved in Tris-HCl buffer, 0.25 μg/mL, final concentration) and 8 μL substrate (DDAO-ol, dissolved in DMSO, 20 μM, final concentration). After pipetting out the residual DMSO in the new path (P5), the prepared enzyme-substrate mixture was immediately injected into the P5 fluidic path. Finally, the top layer was slipped down and to the right, transporting the serial gradient array of inhibitor to the solution of the enzyme-substrate system. As shown in Figure 1c, in the enzyme-substrate system, the enzyme (hPL) could catalyze the substrate (DDAO-ol) to release the fluorescent product DDAO, which led to an increase in the fluorescent intensity in the microchambers. By reacted with the inhibitor, the enzyme-catalyzed reaction was inhibited to stop the increase in fluorescent intensity. After 3 min, the SlipChip was then placed under an Olympus IX83 inverted microscope under the same fluorescence conditions at 10-s intervals to obtain photographs of the individual microchambers. The SlipChip device was then placed under the Olympus IX83 inverted microscope with the same fluorescent condition to obtain the photos of each microchamber. The gray value of the fluorescent photo was analyzed by ImageJ software. The residual activity of hPL was calculated using the following formula: the residual activity (%) = the fluorescence intensity of the test sample/the fluorescence intensity in negative control (DMSO only) × 100%.

### 2.7. Statistical Analysis

All inhibition assays were performed in triplicate (three technical replicates) for each concentration of the inhibitors. IC_50_ and values were calculated using GraphPad Prism 7.0 (GraphPad Software, Inc., La Jolla, CA, USA).

## 3. Results and Discussion

### 3.1. Simulation of the Mixing Time

**Figure 2:**
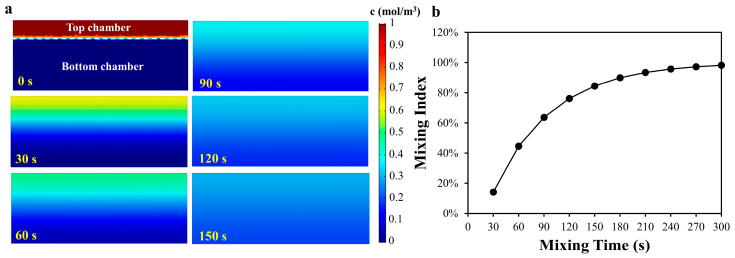
(**a**) The numerical concentration distribution of the cross section of the two microchambers; (**b**) The relationship between mixing index and mixing time.

The mixing of the solutions between two microchambers has great impact on the serial gradient dilution in this proposed microfluidic SlipChip. As the main mixing in two static chambers is based on the diffusion effect, the estimation of the time required for diffusion over a distance equal to the height of the chamber was calculated based on the approximation equation as following:

(1) $$t_{e}\approx \frac{x^{2}}{2D}$$

where x is the mean distance traveled by the diffusing solute in one direction along one axis after elapsed time t, and D is the diffusion coefficient of a solute in free solution. Considering the complex composition of the actual experimental solution, we set D as 5 × 10^−6^ cm^2^/s for the calculation. An estimation of the time required for the ions and molecules to move from the top chamber to the bottom chamber was calculated as 360 s. In order to calculate the appropriate mixing time at the three-dimensional scale, the finite element analysis (FEA) software COMSOL (5.3a, Burlington, MA, USA) was first used to pre-determine the mixing time. The boundary conditions of the simulation can be found in the Supplementary Information. To ensure that the solutions in two microchambers could be completely mixed, the top layer chamber at 200 μm height and the bottom layer chamber at 600 μm height were selected for simulation. To evaluate the degree of mixing, the simulated mixing index (M) is defined as follows [36].

(2) $$M=1-\;\frac{\sqrt{\frac{1}{n}\sum_{i=1}^{n}{\left(c_{i}-c_{m}\right)}^{2}}}{c_{m}}$$

where *c_i_* is the concentration of the point *i*, *c_m_* is the average concentration of all the points, and *n* is the total number of the points in two microchambers. Figure 2 showed the concentration distribution of the cross section of the two microchambers and the mixing index with different mixing time. As shown in Figure 2b, the mixing index increased rapidly with the increase in the mixing time, and increased slowly when the mixing time reached 3 min. However, it could be found from the results that when the mixing time was 5 min, the mixing efficiency still did not reach 100%. In the real-world case, when sliding the SlipChip, an initial velocity will be applied on the liquid in the chamber and the liquid will be also squeezed due to the deformation of the PDMS, which will accelerate the solutions mixing. Considering that the above conditions were difficult to quantify in the simulation, only static liquid simulation was applied to obtain an approximate time point at which the mixing efficiency increases slowly, to provide a reference for the mixing experiment.

### 3.2. Experiment of the Optimization of Mixing Time

**Figure 3:**
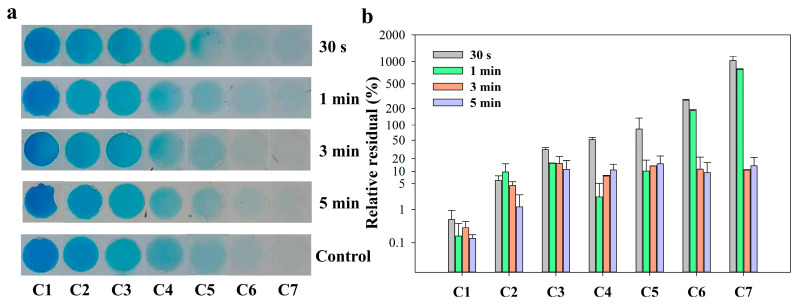
(**a**) Photos of gradient concentration array of blue ink solution in seven microchambers with different mixing time and control group performed in tube; (**b**) The relative residual of saturation of the microchambers area in the photos.

To compare the actual mixing in this SlipChip with the simulation results, blue dye (as solution 1) and PBS (0.01 M, as diluent) were used to optimize the mixing time. The detailed operation process can be found in Section 2.4. All photos of the serial blue dye array with gradient concentrations in microchambers are shown in Figure 3a. It was obvious from the photos that when the mixing time was less than 3 min, the color of the microchambers on the right (C6 and C7) was still light blue, which were darker than the one of the control group. When the mixing time was over 3 min, all the colors of each microchamber were similar to the control group. To better determine the difference between the experimental group and the control group, the relative residual of saturation value of the microchamber area in the photo was selected for comparison. The relative residual was calculated using the formula: $Relative\;residual\;\left(\%\right)=\left|S_{e}-S_{c}\right|/S_{c}\times 100\%,$ where *S_e_* is the saturation value of the experimental group, and S_c_ is the saturation value of the control group. As shown in Figure 3b, no matter the mixing time conditions, the relative residuals in the first two chambers had no significant change between each group. It can be explained that the saturation value of the first two chambers (C1&C2) was supersaturated due to the high concentration of the solutions and changed little. In the rear chambers (C3–C7), when the mixing time was less than 3 min, there was a large relative residual between the experimental group and the control group. The relative residual of each chamber reduced around 10% when the mixing time increased to 3 min. The results suggested that 3 min of mixing time by diffusion was sufficient to allow the blue dye solution and PBS diluent to reach complete mixing. Considering the overall operation time, therefore, a mixing time of 3 min was applied to the following serial dilution experiments. 

### 3.3. Fluorescence Gradient Dilution Test 

**Figure 4:**
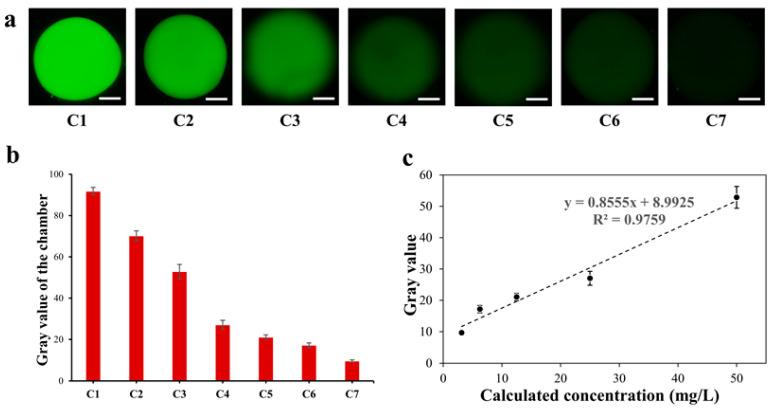
Fluorescence gradient dilution test of microfluidic SlipChip. Error bars show the standard deviations of measurements from at least three separate experiments. (**a**) Fluorescent pictures of rhodamine fluorescent dye in seven microchambers of SlipChip. White scale bar: 500 µm; (**b**) The gray value of the fluorescent pictures in the microchamber of the SlipChip; (**c**) Linear relation diagram of fluorescent dye concentration (3.125 mg/L to 50 mg/L) and gray value. This followed the linear equation: y = 0.8555x + 8.9925, with a correlation of R^2^ = 0.9759.

To ensure the serial dilution ability of this SlipChip for fluorescence detection, a standard fluorescent solution (Rhodamine 6G, 400 mg/L) and DI water were utilized for evaluating the level of fluorescent dilution. According to the operation described in Section 2.5, a series of microchambers containing fluorescent dye solution with seven gradient concentrations was established. Figure 4a showed fluorescent photos of seven microchambers in the proposed SlipChip after serial gradient dilution. The fluorescence intensity decreased with the increase in the dilution ratio of the fluorescent dye solution concentration, and the gray value of the fluorescent photos of each chamber is shown in Figure 4b. The results showed that fluorescent dye solution could be gradient diluted serially on the SlipChip, and the SlipChip had a good fluorescence detection performance. Figure 4c shows that there was a good linear relationship between the fluorescence gray value and the calculated concentration in the microchambers of the sliding chip; when the concentration of the dye ranged from 3.125 mg/L to 50 mg/L, the linear relationship between gray value (y) and concentration (x) could be expressed as y = 0.8555x + 8.9925 (R^2^ = 0.9759). The measured fluorescent intensity was in good agreement with the expected fluorescence intensity, which meant that the proposed SlipChip can produce a fluorescence gradient dilution test. 

### 3.4. Analysis of the IC_50_ of Inhibitors Characterizing Results

**Figure 5:**
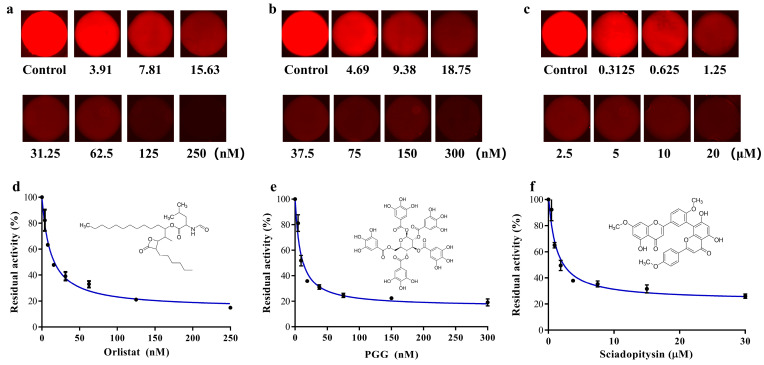
(**a**–**c**) Fluorescence pictures of Orlistat, PGG and sciadopitysin, with seven gradient concentrations after reaction with pancreatic lipase substrate; (**d**–**f**) Dose-inhibition curves of Orlistat, PGG and sciadopitysin against hPL-catalyzed DDAO-ol hydrolysis. Inserts illustrated the chemical structures of Orlistat, PGG and sciadopitysin. Error bars showed the standard deviations of measurements from at least three separate experiments.

According to the operation described in Section 2.6, the three inhibitor (Orlistat, PGG and sciadopitysin) solutions with seven gradient concentrations were established in the microchambers of the SlipChip. The inhibitors at gradient concentrations were reacted with the enzyme-substrate systems in the microchambers of the SlipChip. The solution containing only DMSO was used as a control group to react with the incubation system. The microfluidic SlipChip was placed under the fluorescence microscope to capture fluorescent photos. The fluorescent photos of the three groups of concentration gradient inhibitor solutions after reaction with the enzyme substrate are shown in Figure 5a–c. From the results we found that, in the absence of inhibitors, the specific substrate DDAO-ol would react with hPL and could be readily hydrolyzed to release DDAO, which triggered a dramatic increase in fluorescent signal in the microchambers. However, when the inhibitors were added, a significant decrease in fluorescence intensity could be observed, which was due to the ability of the inhibitor to bind to hPL, thus reducing the reaction of hPL with the specific fluorescent substrate DDAO-ol, resulting in less DDAO production. The known hPL inhibitors (Orlistat, PGG, and sciadopitysin) strongly inhibited hPL-catalyzed DDAO-ol hydrolysis in a dose-dependent manner. The IC_50_ values of these three agents were calculated as 11.69 nM, 8.22 nM, and 0.80 μM, for Orlistat, PGG, and sciadopitysin, respectively, using this proposed microfluidic SlipChip, which was close to the IC_50_ values that we previously detected by conventional methods (7.98 nM, 7.59 nM, and 0.75 μM, respectively). 

The results showed that the proposed SlipChip could provide unique advantages for characterizing enzyme inhibitor concentration. Compared with 96-well plate and commercial microplate reader-based methods, the SlipChip has the advantages of portability, less reagent consumption and less experimental cost. In addition, the SlipChip can also be used to characterize and screen inhibitors or agonists of other kinds of enzymes. A serial solution array with gradient concentrations was established in the microchamber of the SlipChip with simple slipping operations, and these solutions can be manipulated in parallel to merge with a second set of reagents to perform a screening test. The entire process does not require a precise fluidic control instrument, and it can be accomplished by simple manual operations. Furthermore, the SlipChip can also realize a wider range of screening by adjusting the initial inhibitor concentration and the height or number of microchambers of the SlipChip according to the experimental and testing requirements.

## 4. Conclusions

In summary, we have developed a SlipChip that can generate serial arrays with gradient concentrations and introduce an additional reagent to the solutions in parallel through simple sliding operations. Without cumbersome manual pipetting steps or complex fluidic handling systems, the proposed SlipChip can provide a precise dilution ratio by controlling the number and height difference of microchambers. Under optimized conditions, we successfully performed an enzyme inhibition assay and plotted the dose-inhibition curves on the SlipChip for Orlistat, PGG and sciadopitysin. The IC_50_ values of each inhibitor against hPL were calculated and the results were in agreement with previously reported results, suggesting that the SlipChip could be successfully applied to drug screening with the advantages of less reagent consumption and less experimental cost. Furthermore, this proposed SlipChip system has a great potential for biological activity assays, drug screening and clinical diagnostics.

## Data Availability

All data are presented in the main text of this manuscript.

## Supplementary Materials

The following supporting information can be downloaded at: https://www.mdpi.com/article/10.3390/bios13020274/s1, Figure S1: The fabrication processing of microfluidic SlipChip; Figure S2 and Table S1: The detailed microfluidic SlipChip design parameters.
