# Supplementary material for: Construction and Manipulation of Serial Gradient Dilution Array on a Microfluidic Slipchip for Screening and Characterizing Inhibitors against Human Pancreatic Lipase
Source: Biosensors (Basel). 2023 Feb 15;13(2):274. doi: 10.3390/bios13020274 (PMC9954273; doi:10.3390/bios13020274)
Supplement: Supplementary file 1 [file biosensors-13-00274-s001.zip › biosensors-2160776-supplementary.pdf]

biosensors

*Supplementary Information*

# Construction and Manipulation of Serial Gradient Dilution Array on a Microfluidic Slipchip for Screening and Characterizing Inhibitors against Human Pancreatic Lipase

**Junqiang Yang [1,†], Yanyan Deng [2,†], Min Zhang[2,3] , Shilun Feng[4], Sheng Peng[5] , Shijia Yang[4], Peirong Liu[1,*], Gaozhe Cai[4,*], Guangbo Ge[2]**

[1] Department of Anesthesiology, Seventh People's Hospital of Shanghai University of TCM, Shanghai, China;
[2] Shanghai Frontiers Science Center of TCM Chemical Biology, Institute of Interdisciplinary Integrative Medicine Research, Shanghai University of Traditional Chinese Medicine, Shanghai, China;
[3]Key Laboratory of Xinjiang Phytomedicine Resource and Utilization, Ministry of Education, Pharmacy School of Shihezi University, Xinjiang, China
[4] State Key Laboratory of Transducer Technology, Shanghai Institute of Microsystem and Information Technology, Chinese Academy of Sciences, Shanghai, 200050, China;
[5] Department of Anesthesiology, Longhua Hospital Shanghai University of TCM, Shanghai, China;
† These authors contributed equally to this work.

* Correspondence: lpeir@126.com (P.L,); gaozhe@mail.sim.ac.cn (G.C.)

**Citation:** Yang, J.; Deng, Y.; Zhang, M.; Feng, S.; Peng, S.; Yang, S.; Liu, P.; Cai, G.; Ge, G. Construction and Manipulation of Serial Gradient Dilution Array on a Microfluidic Slipchip for Screening and Characterizing Inhibitors against Human Pancreatic Lipase. *Biosensors* **2023**, *13*, 274. https://doi.org/10.3390/bios13020274

Received: 6 January 2023
Revised: 10 February 2023
Accepted: 13 February 2023
Published: 15 February 2023

**Copyright:** © 2023 by the authors. Licensee MDPI, Basel, Switzerland. This article is an open access article distributed under the terms and conditions of the Creative Commons Attribution (CC BY) license (https://creativecommons.org/licenses/by/4.0/).

## 1. The fabrication processing of microfluidic slipchip

A silicon wafer was first prepared by washing with acetone and DI water. Then, 1 mL of silicone oil was dropped on the silicon wafer, followed by spinning at 3000 rpm for 50 s. After that, the cured PDMS bottom layer from a 3D printed mold was stamped onto the silicone wafer for 1 min to form a silicone oil film. Then, the PDMS bottom layer with silicone oil film was peeled from the wafer and assembled with the punched PDMS top layer. After assembling, the SlipChip was then placed horizontally for 10 min to make the silicon oil layer uniform. The fabrication processing of microfluidic SlipChip is shown in Figure S1.

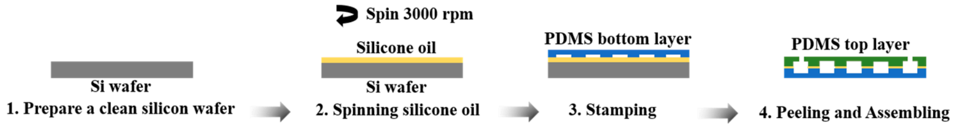


**Figure S1**. The fabrication processing of microfluidic SlipChip.

## 2. The detailed microfluidic slipchip design parameters

The microfluidic SlipChip consists of two opposing microfluidic layers that have microfabricated microchambers and fluidic ducts on the contact surface. The top PDMS layer consists of four rows of microchambers (diameter: 2 mm, height: 200 μm) and ducts (3 mm*1 mm*0.2 mm) with of the same height. The bottom PDMS layer consists of five rows of microchambers with different heights (200 μm and 600 μm) and the same diameter (2 mm). The detailed design parameters are shown in Figure S2 and Table S1.

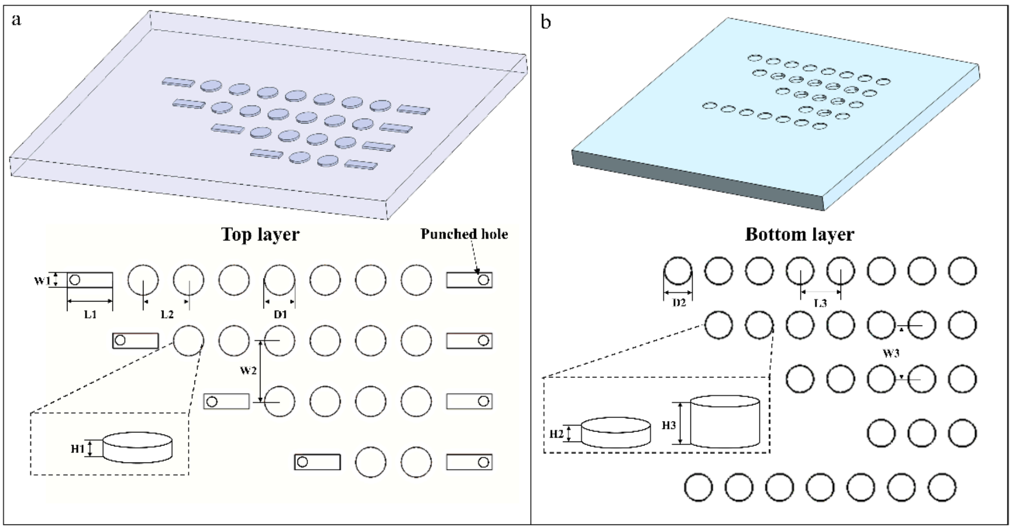


**Figure S2.** The design of the microfluidic SlipChip.

| | | | |
|---|---|---|---|
| $L_1$ | 3 mm | $L_3$ | 3 mm |
| $W_1$ | 1 mm | $D_2$ | 2 mm |
| $H_1$ | 200 μm | $W_3$ | 4 mm |
| $D_1$ | 2 mm | $H_2$ | 200 μm |
| $L_2$ | 3 mm | $H_3$ | 600 μm |
| $W_2$ | 4 mm | | |

**Table S1.** The detailed parameters of the microfluidic SlipChip.

## 3. Boundary conditions of the simulation

In the simulation of this work, the diffusion coefficient was set as $5\times10^{-10}$ $m^2/s$ and the concentrations of the solution in the top chamber and bottom chamber were set as 1 mol/$m^3$ and 0 mol/$m^3$, respectively. The solution was set as water with a fluid viscosity of $1\times10^{-3}$ Pa·s and a density of $1\times10^3$ kg/$m^3$. The simulation is on a three-dimensional scale and the heights of the top layer chamber and bottom layer chamber were set as 200 μm and 600 μm, respectively. The diameter of the chambers was set as 2 mm.
